# Network analysis of miRNA targeting m6A-related genes in patients with esophageal cancer

**DOI:** 10.7717/peerj.11893

**Published:** 2021-07-29

**Authors:** Lili Li, Rongrong Xie, Qichun Wei

**Affiliations:** 1 Department of Radiation Oncology, The Second Affiliated Hospital, Zhejiang University School of Medicine, Hangzhou, China; 2 Department of Medical Oncology, The First Affiliated Hospital of Wenzhou Medical University, Wenzhou, China

**Keywords:** Esophageal cancer, HNRNPC, N6-methyladenosine, Overall survival, Prognosis

## Abstract

**Background:**

We investigated the miRNA-m6A related gene network and identified a miRNA-based prognostic signature in patients with esophageal cancer using integrated genomic analysis.

**Methods:**

We obtained expression data for m6A-related genes and miRNAs from The Cancer Genome Atlas (TCGA) and Gene Expression Omnibus (GEO) datasets. Survival analysis was conducted to identify potential prognostic biomarkers. LASSO Cox regression was performed to construct the overall survival (OS) associated prediction signature. We used the Kaplan–Meier (K–M) curve and receiver operating characteristic (ROC) curves to explore the signature’s efficiency and accuracy. Interactions between the m6A-related genes and miRNAs were identified in starBase3.0 and used to construct the miRNA-m6A related gene network.

**Results:**

We found that HNRNPC, YTHDF, ZC3H13, YTHDC2, and METTL14 were dysregulated in esophageal cancer tissues. Multivariate Cox regression analysis revealed that HNRNPC may be an independent risk factor for OS. Five hundred twenty-two potential upstream miRNAs were obtained from starBase3.0. Four miRNAs (miR-186, miR-320c, miR-320d, and miR-320b) were used to construct a prognostic signature, which could serve as a prognostic predictor independent from routine clinicopathological features. Finally, we constructed a key miRNA-m6A related gene network and used one m6A-related gene and four miRNAs associated with the prognosis. The results of our bioinformatics analysis were successfully validated in the human esophageal carcinoma cell lines KYSE30 and TE-1.

**Conclusion:**

Our study identified a 4-miRNA prognostic signature and established a key miRNA-m6A related gene network. These tools may reliably assist with esophageal cancer patient prognosis.

## Introduction

Esophageal cancer is one of the most aggressive malignant tumors: it is the ninth most common cancer and the sixth most common cause of cancer-related deaths in the world (Lagergren et al., 2017). Surgery is the main treatment modality for resectable esophageal cancer (Shen et al., 2015), however, treatment failure may occur due to local recurrence and distant metastasis. The prognosis is poor for late-stage patients despite improved surgical treatment and multimodality therapies for patients (Chen et al., 2019). Therefore, it is critical to identify novel prognostic biomarkers and molecular targets for esophageal cancer patients.

N6-methyladenosine (m6A) is a universal modification of eukaryote RNA molecules. It plays a critical role in RNA processing by acting as writers, readers, and erasers (Yang et al., 2018). Growing evidence has demonstrated the correlation between m6A and human cancers, including breast cancer, hepatocellular carcinoma, lung cancer, and pancreatic cancer (Ma et al., 2017; Taketo et al., 2018; Wanna-Udom et al., 2020; Zhang et al., 2016). The literature has also indicated that m6A-related genes might serve as novel prognostic biomarkers for different cancers, and that the m6A-related risk score may assist with risk assessment and prognostic stratification (Guan et al., 2020; Zhang et al., 2020). Recently, a few studies have been conducted to determine the role of m6A methylation regulators in esophageal cancer. Guo et al. (2020) reported that m6A and its regulator, HNRNPA2B1, were significantly elevated in esophageal cancer. They also reported that HNRNPA2B1 played a carcinogenic role in esophageal cancer cell progression. Xu, Pan & Pan (2020) constructed and validated an m6A RNA methylation regulators-based signature as an independent prognostic predictor for esophageal cancer patients.

MicroRNAs (miRNAs) are a class of non-coding RNAs with potent regulatory functions in cancer cell proliferation, differentiation or apoptosis (Baltimore et al., 2008; Karrich et al., 2013). Abnormally expressed miRNAs have been detected in many human cancers, such as prostate cancer, gastric cancer, bladder cancer, lung cancer and esophageal cancer (El Bezawy et al., 2017; He et al., 2017; Hummel et al., 2014; Wang et al., 2018; Xu et al., 2017). A growing body of evidence has shown that miRNAs play an important role in the pathogenesis, biological behavior, and multi-drug resistance of esophageal cancer (Chang et al., 2018; Dias et al., 2018; Fang, Fang & Hu, 2012; He et al., 2019; Hummel et al., 2014; Sharma, Saini & Sharma, 2017). Some miRNAs (*e.g*., miR-485-5p, miR-200c, miR-183, miR-224 and miR-624-3p) can regulate the proliferation and invasion of esophageal cancer cells and can also affect the sensitivity of esophageal cancer cells to radiotherapy and chemotherapy (Chang et al., 2018; Han et al., 2019; He et al., 2019; Yang et al., 2014; Zheng et al., 2017). Other miRNAs (*e.g*., miR-18a, miR-155 and miR-21) may be used as biomarkers for the diagnostic and prognostic evaluation of esophageal cancer (Hirajima et al., 2013; Liu et al., 2012; Tanaka et al., 2013). Furthermore, recent studies have highlighted the significant value of miRNA signatures in predicting esophageal cancer prognosis (Chen et al., 2018; Xue et al., 2021). Though miRNAs’ contributions to the development of esophageal cancer has been extensively studied, there have been few studies on their mechanisms for targeting m6a-related genes in esophageal cancer. A better understanding of miRNAs’ network relationships and m6A-related genes may help identify biomarkers as potential therapeutic targets.

The Cancer Genome Atlas (TCGA) database (https://cancergenome.nih.gov/) provided us with complicated clinical characteristics and cancer genomics (Kim et al., 2017). In the present study, we found that m6A-related genes were frequently differentially expressed in esophageal cancer. Heterogeneous nuclear ribonucleoprotein C (HNRNPC), an RNA binding protein, was overexpressed in esophageal cancer tissues and cell lines. We also found that a high level of HNRNPC was associated with poor prognosis in esophageal cancer patients. We obtained seven predicted upstream miRNAs from the starBase dataset (http://starbase.sysu.edu.cn/) and established a four-miRNA signature to be used as a potential prognostic biomarker of esophageal cancer. We evaluated the signature using the Gene Expression Omnibus (GEO) microarray for the validation cohort. Finally, we constructed a network of key miRNAs targeting m6A-related genes and the bioinformatics prediction was further validated in the human esophageal carcinoma cell lines KYSE30 and TE-1.

## Materials & methods

### Data from TCGA and GEO datasets

We downloaded m6A-related gene expression profiling data for 162 esophageal cancer samples and 11 normal samples from the TCGA repository (https://portal.gdc.cancer.gov/). We predicted the upstream miRNAs for each m6A-related gene using the online tool starBase3.0 (http://starbase.sysu.edu.cn/). miRNA expression profiles for 185 esophageal cancer samples and 13 normal samples were also obtained from TCGA. Patient inclusion criteria included: (1) the availability of complete survival data and (2) an esophageal cancer histology type. Patients with a survival time of less than 100 days were excluded from our study. We enrolled 164 esophageal cancer patients in the survival analysis. We included 107 cases with full clinicopathological data for further Cox proportional hazard regression analysis. miRNA microarray expression profiling data and the clinical data for 119 patients with esophageal cancer were obtained from the GEO microarray dataset (GSE43732) as validation cohort data.

### Screening of differentially expressed genes

We analyzed the differentially expressed m6A-related genes and miRNAs between esophageal cancer samples and matched healthy tissues using the “limma” package in R (Ritchie et al., 2015). The T-test in the limma package was used to calculate the differentially expressed genes (DEGs) in the senescent and quiescent. A *P*-value < 0.05 was considered to be significant.

### Identification of prognosis-related genes

The univariate cox regression test and Kaplan–Meier (K–M) survival analysis were performed to screen out the potential overall survival (OS) relevant genes. A *P*-value less than 0.05 was considered to be statistically significant. We used multivariate cox analysis to evaluate the contribution of the selected genes. The analysis was conducted using the R package of survival.

### Construction and evaluation of the miRNA-based prognostic signature

We used the “glmnet” package to perform LASSO analysis (https://CRAN.R-project.org/package=glmnet) on the selected miRNAs. The prognostic miRNA-based signature was designed using a risk scoring method with the following formula:

\rm RickScore = {\sum_{i=1}^N} (\rm exp*coef)

in which exp was the expression value of the gene and coef was the coefficient of miRNA generated from the LASSO-Cox regression analyses. The median risk score was set as the cutoff point, according to which the esophageal cancer patients were divided into low-risk and high-risk groups. We then measured the survival difference between the high-risk and low-risk groups stratified by the OS-related miRNA prediction model. The area under the curve (AUC) of the receiver operating characteristic (ROC) curve was calculated to assess the efficacy and accuracy of the OS classifier. We conducted the univariate and multivariate Cox proportional hazards analyses to compare the relative prognostic value of the miRNA-based signature with that of different clinicopathological features.

### Cell culture and transfection

Human normal esophageal epithelial cell line (HEEC) and esophageal carcinoma cell lines (KYSE30 and TE-1) were purchased from American Type Culture Collection (ATCC, Manassas, VA, USA). Both cell lines were cultured in RPMI 1640 medium (Gibco, Waltham, MA, USA) supplemented with 10% fetal bovine serum (FBS, Belize, Gibco, Waltham, MA, USA), 100 U/ml penicillin and 100 mg/ml streptomycin (Invitrogen, Carlsbad, CA, USA). Mir-186 mimics, mimic-NC, sh-HNRNPC (sh-HNRNPC#1 and sh-HNRNPC#2) and sh-NC were constructed by RiboBio (Guangzhou, China). Esophageal carcinoma cell lines were placed into six-well plates until cell confluence was approximately 70%. The miR-186 mimics, mimic-NC, sh-HNRNPC, or sh-NC were transfected into esophageal carcinoma cell lines according to the Lipofectamine 2000 transfection reagent (Invitrogen, Carlsbad, CA, USA).

### Quantitative real-time PCR (qRT-PCR)

Total RNA was extracted from cells using TRIzol reagent (Takara, Japan). Messenger RNA (mRNA) and miRNA were reverse-transcribed into complementary DNA with the cDNA Synthesis Kit (ABM, Vancouve, Canada). qRT-PCR was performed with a SYBR Green PCR kit (Takara, Kusatsu, Japan) and the Bio-Rad Real-Time PCR System (Bio-Rad, Hercules, CA, USA). GAPDH was treated as the internal control gene for HNRNPC, which U6 was the endogenous control gene for miRNAs. Primer information is presented in Table 1. The relative expression level was analyzed using the 2^−ΔΔCt^. All assays were performed in triplicate.

**Table 1:** Primers used in qRT-PCR.

Name	Primer sequences (5′→3′)
miR-186	F 5-CGACGCGTCGGGTTTACAGAACACCCATCA-3
miR-320b	F 5-TCCGAAACGGGAGAGTTTTGG-3
miR-320c	F 5-AAAAGCTGGGTTGAGAGGGT-3
miR-320d	F 5-ACACTCCAGCTGGGAAAAGCTGGGTTGAGA-3
HNRNPC	F 5-AGAACCCGGGAGTAGGAGAC-3
	R 5-TCTCACAAAGCCGAAAACAA-3
GAPDH	F 5-CAGCTAGCCGCATCTTCTTTT-3
	R 5-GTGACCAGGCGCCCAATAC-3
U6	F 5-CTCGCTTCGGCAGCACA-3
	R 5-AACGCTTCACGAATTTGCGT-3

### MTT assay

Cell proliferation was evaluated by the 3-(4,5-dimethylthiazol-2-yl)-2,5-diphenyl-tetrazolium bromide (MTT) assay. Cells were plated in 96-well plates at a density of 4,000 cells per well in 100 μl of complete medium and cultured for 24 h. The MTT solution (5 mg/ml, 20 μl) was added to each well and incubated for 4 h at 37 °C. The media was then removed and 100 μl DMSO was added and thoroughly mixed for 15 min. The relative number of surviving cells was assessed by measuring the optical density (OD) of cell lysates at 490 nm. Each group contained five wells. All assays were performed in triplicate.

### Cell migration and invasion assays

The ability of cell migration and invasion was detected with polycarbonate membrane Boyden chambers in a transwell apparatus (Costar, Irvine, CA, USA). We evenly covered the upper surface of the membrane with 50 μL of 1 mg/ml Matrigel (Sigma, St. Louis, MO, USA), incubated it for 1 h at 37 °C, and hydrated it in fetal bovine serum (FBS) for 2 h before use for the invasive assay. The cells were digested and added into the top chamber at the cell density of 3 × 10^4^ cells/ml. The lower chamber was filled with cell culture medium containing 20% FBS and was incubated at 37 °C for 36 h. After incubation, the invasive cells were fixed, stained, and counted under a microscope. In the cell migration assay, the same procedures were conducted without Matrigel on the membrane. Three independent experiments were performed.

### Luciferase reporter assay

We inserted the 3′-UTR of wild-type HNRNPC (HNRNPC-WT) with mutations in the putative binding site downstream of the frefly luciferase reporter into the psiCHECK-2 vector (Promega, Madison, WI, USA). The corresponding mutant construct was created by mutating the seed regions of the miR-186 binding site. This site was named 3′-UTR HNRNPC-MUT. HEK293 cells were co-transfected with miR-186 mimics or the NC control, then combined with either HNRNPC-WT or HNRNPC-MUT reporter vectors for 48 h. Cells were harvested using the dual Glo™ Luciferase Assay System (Promega, Madison, WI, USA) to measure luciferase activity. Renilla activity was used as an internal control. Experiments were conducted independently at least three times.

### Statistical analysis

Data were shown as mean ± standard deviation (SD). K–M survival analysis and univariate/multivariate Cox proportional hazards analyses were used to compare the two groups of patients. HR and 95% confidence intervals (CIs) were calculated. All statistical analyses were performed by SPSS 22.0 software (IBM Corp., Armonk, NY, USA). *P* < 0.05 was considered to be statistically significant.

## Results

### The expression pattern of m6A-related genes in esophageal cancer

**Figure 1:**
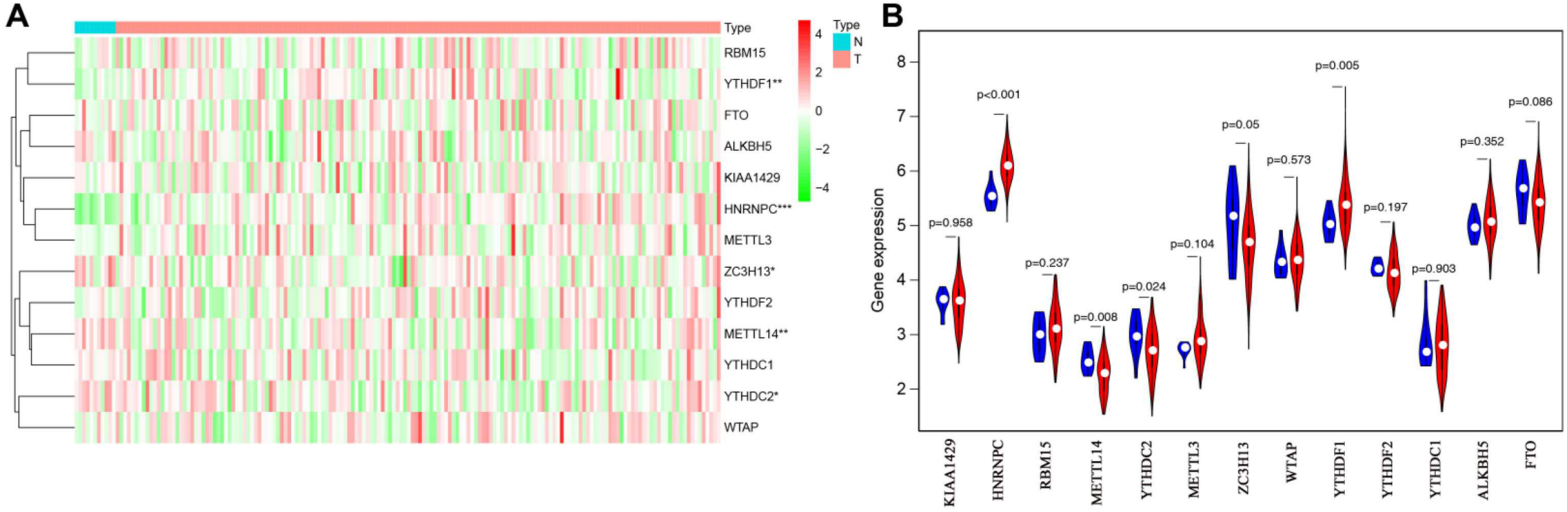
Bioinformatics analysis of the expression patterns of m6A-related genes in esophageal cancer. (A) The heatmap indicated the expression of m6A-related genes in esophageal cancer (T, *n* = 162) and normal tissues (*N*, *n* = 11) based on the data from TCGA. *P < 0.05; **P < 0.01; ***P < 0.001. (B) Volcano plot showed gene expression profiles in esophageal cancer (red, *n* = 162) and normal tissues (blue, *n* = 11).

To explore the expression patterns of m6A-related genes in esophageal cancer, we first extracted and analyzed the expression data of 13 known m6A-related genes from the TCGA database. HNRNPC and YTHDF1 were significantly upregulated in tumor tissues compared with corresponding normal tissues, while ZC3H13, YTHDC2 and METTL14 notably decreased (Figs. 1A and 1B). These results suggested that m6A modification may affect esophageal cancer development.

### Prognostic analysis of m6A-associated genes in esophageal cancer

**Figure 2:**
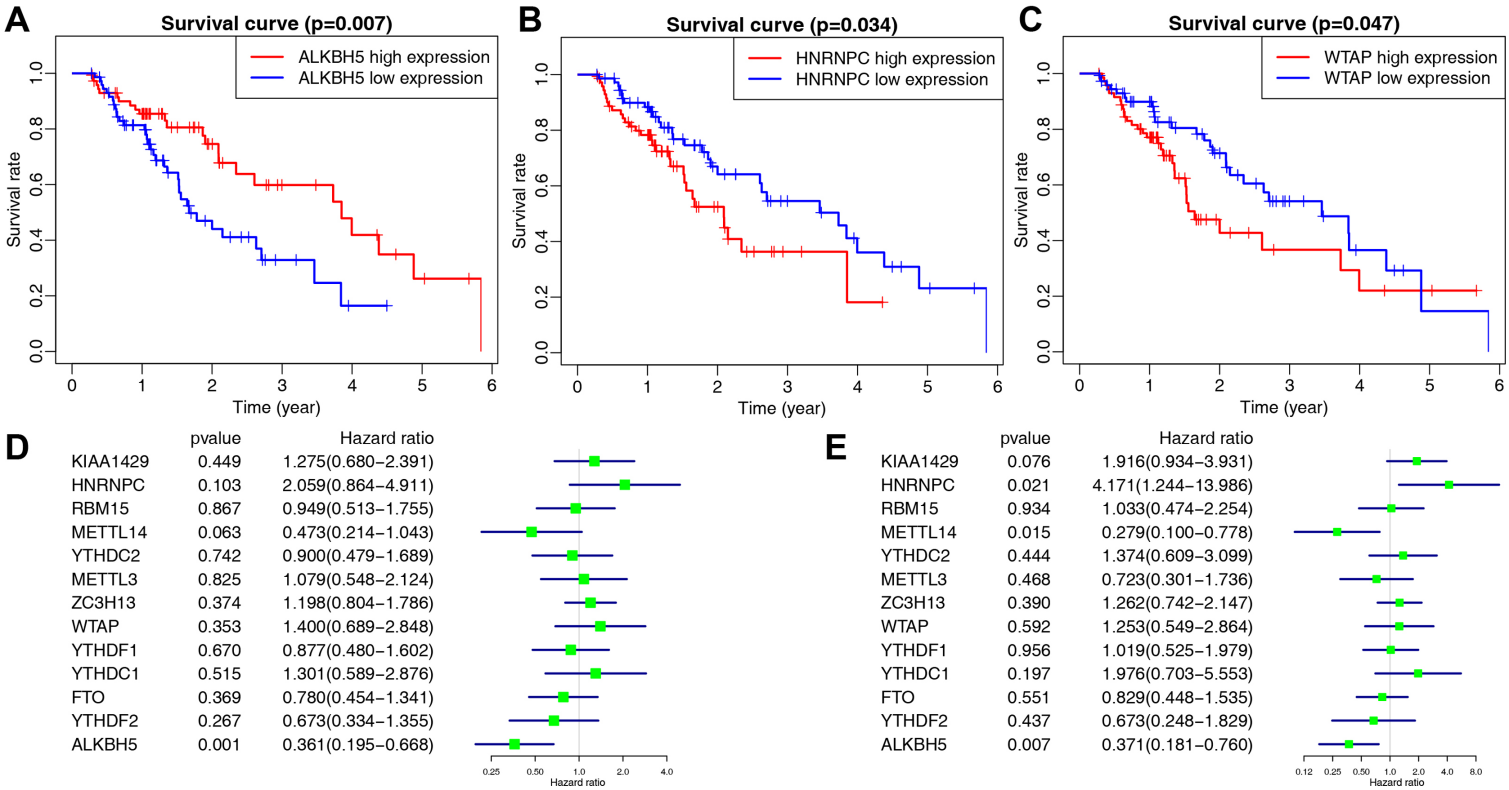
The correlation between the expression levels of m6A-related genes and overall survival (OS) rates in esophageal cancer patients (n = 162). (A–C) Kaplan–Meier OS curve based on ALKBH5 expression (A), HNRNPC expression (B) and WTAP expression (C) in TCGA dataset. (D–E) Univariate (D) and multivariate (E) analyses of m6A-related genes associated with OS.

The main clinicopathological characteristics of esophageal cancer patients are summarized in Table 2. We analyzed the correlation between m6A-related gene expression and clinical follow-up data using K–M survival analysis to investigate the prognostic role of m6A-associated genes in esophageal cancer. Patients were divided into the high- or low-expression groups based on the best cut-off value. The results showed that the low expression of ALKBH5 and the high expression of HNRNPC or WTAP were the best predictors for poor OS rates in esophageal cancer patients (Figs. 2A–2C).

**Table 2:** The main clinicopathological characteristics of the 107 esophageal cancer patients.

Parameter	*N* = 107(%)
Gender	
Male	92 (86.0%)
Female	15 (14.0%)
Age	
≤65	75 (70.1%)
>65	32 (29.9%)
Grade	
G1	14 (13.1%)
G2	60 (56.1%)
G3	33 (30.8%)
Stage	
I	16 (15.0%)
II	56 (52.4%)
III	30 (28.0%)
IV	5 (4.6%)
T	
T1	16 (15.0%)
T2	30 (28.0%)
T3	58 (54.2%)
T4	3 (2.8%)
N	
N0	54 (50.5%)
N1	42 (39.2%)
N2	6 (5.6%)
N3	5 (4.7%)
M	
M0	102 (95.3%)
M1	5 (4.7%)

We then performed univariate and multivariate Cox regression analyses to explore whether these genes were associated with the prognosis of esophageal cancer patients. The results of univariate cox regression revealed that ALKBH5 (*P* = 0.001) was a protective gene for esophageal cancer (Fig. 2D). Multivariate Cox regression revealed that both ALKBH5 (*P* = 0.006) and METTL14 (*P* = 0.015) were protective genes for OS. It also revealed that HNRNPC (HR = 4.171, *P* = 0.021, 95% CI [1.244–13.986]) may be an independent risk factor for OS (Fig. 2E). We focused on the m6A-related gene HNRNPC and its role in esophageal cancer in light of our analysis of m6A-related gene expression levels and the prognostic values above.

### Constructing and validating the miRNA-based prognostic signature

**Figure 3:**
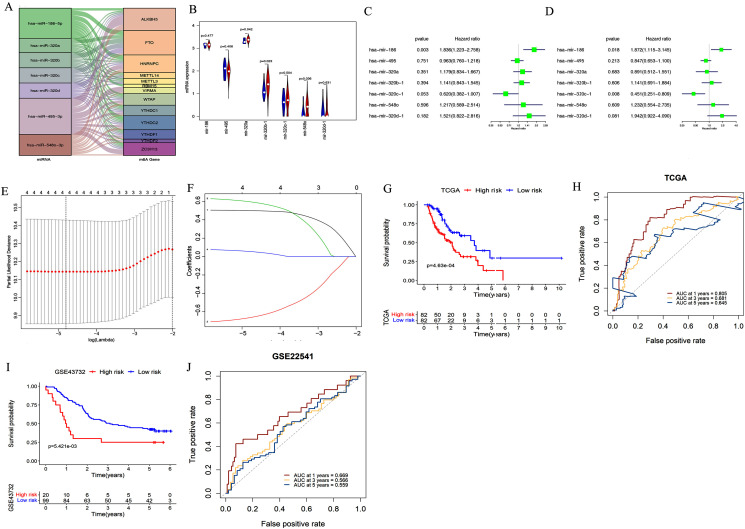
Construction and validation of the miRNA-based prognostic signature. (A) The relationship between the 13 target genes and their corresponding miRNAs was shown. (B) Volcano plot showed miRNA expression profiles in esophageal cancer (red, *n* = 185) and normal tissues (blue, *n* = 13). (C–D) Univariate (C) and multivariate (D) analyses of seven miRNAs associated with overall survival (OS) rates in esophageal cancer patients (*n* = 164). (E–F) The process of using four miRNAs to build the signature. (G, I) Kaplan–Meier OS curves for patients assigned to high-risk and low-risk groups based on the risk score in the TCGA (G) and GEO (I) datasets. (H, J) The receiver operating characteristic (ROC) curves of the risk signature in the TCGA (H) and GEO (J) datasets.

We used starBase 3.0 to assess potential upstream miRNAs targeting m6A-related genes. A total of 522 miRNAs were obtained and the number of m6A-related genes targeted by each miRNA was calculated. The top seven miRNAs with the largest number of target genes were then determined. The relationships between the 13 target genes and their corresponding miRNAs are shown in Fig. 3A. We also explored these miRNAs’ expression patterns in the TCGA dataset. As shown in Fig. 3B, hsa-mir-320b-1 (*P* = 0.023), hsa-mir-548o (*P* = 0.036), and hsa-mir-320a (*P* = 0.042) were upregulated in esophageal cancer tissues compared with normal esophageal tissues.

We further evaluated the prognostic value of these seven miRNAs in esophageal cancer through univariate and multivariate cox regression analysis. The results of univariate cox regression analysis indicated that miR-186 (*P* = 0.003) had prognostic value (Fig. 3C). Multivariate cox regression demonstrated that miR-186 (*P* = 0.018) and miR-320c (*P* = 0.008) may be independent prognostic indicators (Fig. 3D). We used LASSO Cox regression analysis to determine the most prognostic miRNAs among esophageal cancer patients. Finally, four miRNAs (miR-186, miR-320c, miR-320d, miR-320b) were used for the construction of the prognostic signature according to the minimum criteria and the coefficients (Figs. 3E & 3F).

Risk scores for each sample in the TCGA data set were determined using the coefficients derived from the LASSO algorithm. The patients were classified into low- or high-risk groups on the basis of the median risk score. Kaplan–Meier survival curves indicated that patients with higher risk scores (*n* = 82) had significantly shorter OS than those with lower risk scores (*n* = 82, *p* < 0.001, Figu. 3G). A time-dependent ROC curve was performed to evaluate the sensitivity and specificity of the signature. The four-miRNA signature’s AUC values were 0.805, 0.681, and 0.645, respectively, for an OS of 1, 3, and 5 years (Fig. 3H). This indicates that our model could be used to predict esophageal cancer patient survival.

In addition, the accuracy of the risk scoring model was verified using the test set of esophageal cancer cohort in the GEO dataset. We calculated the risk scores for esophageal cancer patients according to the expression levels of miR-186, miR-320c, miR-320d, and miR-320b in the GEO microarray dataset. Patients were divided into high-risk or low-risk groups based on the median risk score of the cohort. Kaplan–Meier analysis showed that patients in the high-risk group had significantly poorer OS than those in the low-risk group (*P* < 0.001; Fig. 3I). ROC curve analysis also showed that our prognostic signature for 1 year had the best predictive performance, which was consistent with the results of TCGA database (AUC values of 1-year (0.669), 3-year (0.566), and 5-year (0.559); Fig. 3J).

### Independence of the miRNA-based signature from other clinicopathological factors

**Figure 4:**
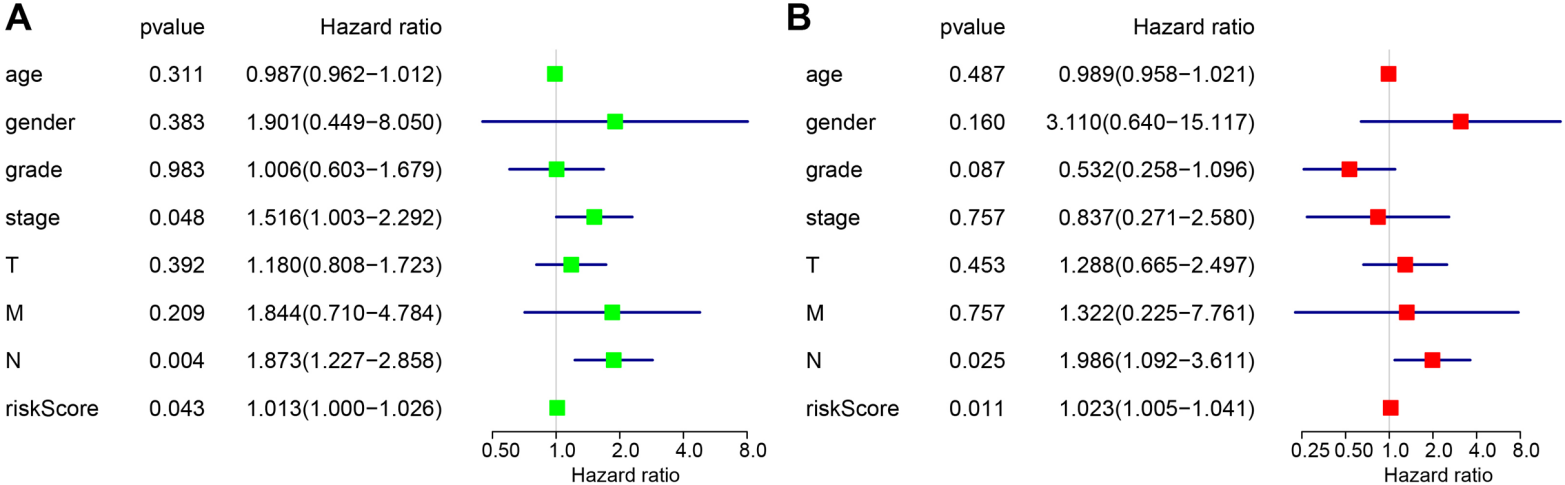
Univariate (A) and multivariate (B) Cox regression analyses of the association between clinicopathological factors (including the risk score) and overall survival of esophageal cancer patients (*n* = 107).

The following clinicopathological characteristics were considered: age, gender, T staging, lymph node status, metastasis, and staging. Univariate and multivariate cox regression were used to detect the influence of the four-miRNA signature on OS. The lymph node status, stage, and risk score in esophageal cancer patients were associated with OS with univariate analysis (Fig. 4A). Multivariate analysis showed that the four-miRNA signature (HR = 1.023, *P* = 0.011) was an independent prognostic factor for esophageal cancer patients (Fig. 4B).

### Construction of key miRNA-m6A related gene network in esophageal cancer

**Figure 5:**
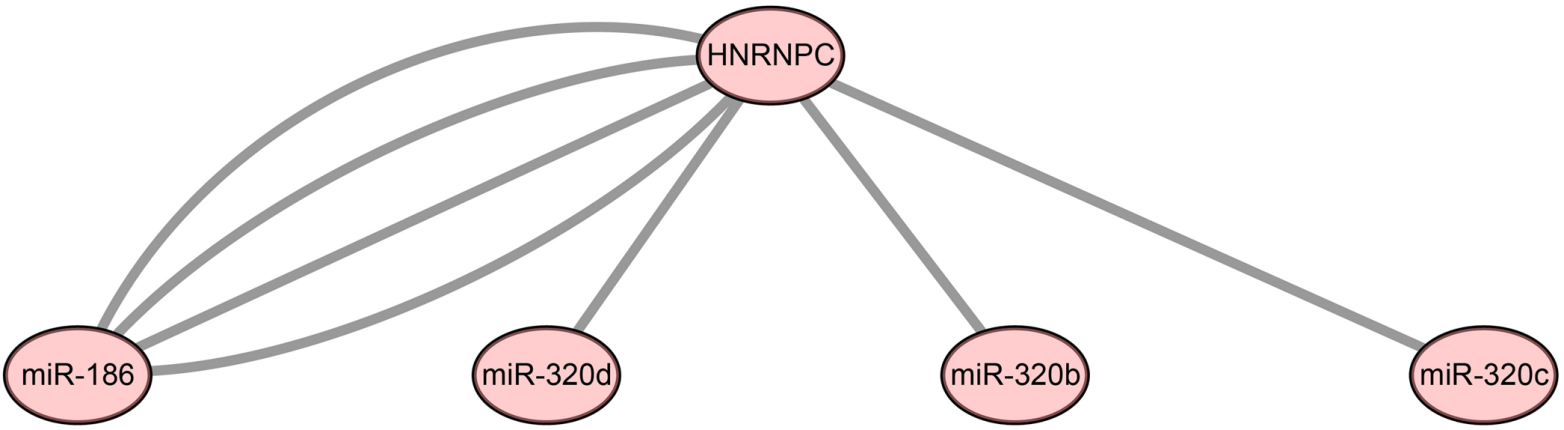
The key miRNA-m6A related gene network in esophageal cancer.

Our results indicated that HNRNPC may be the key m6A-related gene in esophageal cancer. Mir-186, miR-320c, miR-320d, and miR-320b, which were used for the construction of the prognostic signature, may be the key upstream miRNAs in m6A-related genes. We extracted a critical miRNA-m6A related gene network, found that HNRNPC was targeted by these four miRNAs, and then constructed the key miRNA-m6A related gene network (Fig. 5).

### Silencing HNRNPC suppressed proliferation, migration, and invasion of esophageal cancer cells

**Figure 6:**
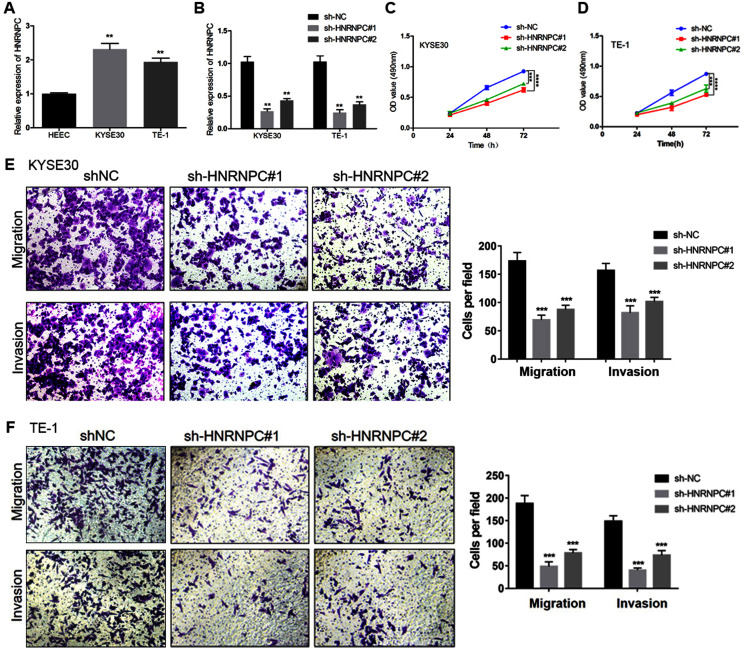
Silencing of HNRNPC suppressed proliferation, migration and invasion of KYSE30 and TE-1 Cells. (A) The mRNA expression levels of HNRNPC in esophageal cancer cell lines (KYSE30 and TE-1) and normal esophageal epithelial cell line HEEC were determined by qRT-PCR. (B) The HNRNPC knockdown efficiency was confirmed at the mRNA levels in KYSE30 and TE-1 cells by qRT-PCR. (C–D) MTT assay was performed to determine the cell proliferation in KYSE30 (C) and TE-1 cells (D). (E–F) Transwell assay was performed to assess the cell migration and invasion capacity in KYSE30 (E) and TE-1 (F) cells. **P < 0.01; ***P < 0.001; ****P < 0.0001.

We further evaluated the expression pattern of HNRNPC in esophageal cancer cells and found that the expression level of HNRNPC in the KYSE30 and TE-1 cell lines was higher than that in the normal esophageal carcinoma epithelial cell line HEEC (Fig. 6A). We transfected a short hairpin RNA (shRNA) targeting HNRNPC into KYSE30 and TE-1 cells to explore the function of HNRNPC. The qRT-PCR confirmed the knockdown efficiency of the two specific shRNAs in both cell lines (Fig. 6B). The MTT assay determined that the knockdown of HNRNPC significantly suppressed esophageal cancer cell proliferation (Figs. 6C & 6D). Furthermore, silencing HNRNPC may also inhibit the migration and invasion of esophageal cancer cells (Figs. 6E and 6F).

### miR-186 targeted HNRNPC and suppressed HNRNPC expression

**Figure 7:**
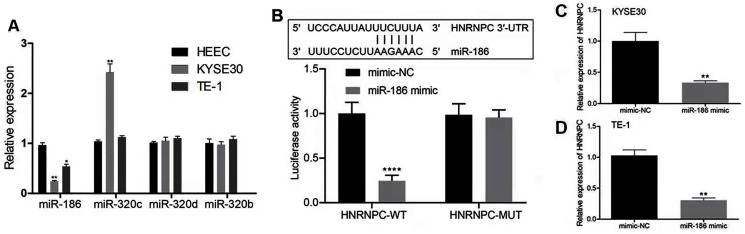
miR-186 targeted HNRNPC and suppressed HNRNPC expression. (A) The relative expression of 4 miRNAs (miR-186, miR-320c, miR-320d, miR-320b) were detected in esophageal cancer cell lines (KYSE30 and TE-1) and normal esophageal epithelial cell line HEEC. (B) Potential binding site of miR-186 in HNRNPC. Detection of miR-186 mimics on luciferase activity of wild-type or mutant HNRNPC by luciferase reporter assay. (C–D) The mRNA expression levels of HNRNPC were assessed by qRT-PCR. *P < 0.05; **P < 0.01; ****P < 0.0001.

We hypothesized that the candidate miRNA would be downregulated in esophageal cancer with a potential binding sequence for the 3′-UTR of HNRNPC. We chose four miRNAs (miR-186, miR-320c, miR-320d, miR-320b) for further validation. miR-186 had low expression levels in both KYSE30 and TE-1 cells compared with HEEC cells (Fig. 7A). Therefore, we chose miR-186 for further studies. The luciferase reporter assay showed that miR-186 mimics significantly reduced the luciferase activity of the reporter gene in HNRNPC-WT compared with that of the negative control (*P* < 0.001) (Fig. 7B). The miR-186 mimics’ regulatory effect was inhibited when the predicted binding site in HNRNPC was mutated. KYSE30 or TE-1 cells treated with miR-186 mimics also decreased HNRNPC expression (Figs. 7C and 7D). These results together indicated that HNRNPC was a direct target of miR-186.

## Discussion

M6A is the most common modification of human mRNA and is considered to be a new epigenetic regulatory layer for mRNA processing, translation and stability (Niu et al., 2013; Patil, Pickering & Jaffrey, 2018). Emerging studies have shown that dysregulation of m6A modification is closely related to various physiological and pathological phenomena in humans including obesity, immune disorders and cancers (Cai et al., 2018; Cui et al., 2017; Chen et al., 2015; Rong et al., 2019). Mounting evidence has also demonstrated that m6A-related genes play crucial roles in the initiation and progression of human cancers. METTL3 has been shown to promote the proliferation and mobility of gastric cancer cells (Lin et al., 2019). YTHDF1 may have oncogenic roles in colorectal cancer (Nishizawa et al., 2018). METTL14 was found to inhibit hematopoietic stem/progenitor differentiation and promote leukemogenesis *via* mRNA m6A modification (Weng et al., 2018). Recently, HNRNPA2B1 was shown to function as an oncogenic factor in promoting esophageal cancer progression by the up-regulation of fatty acid synthesis enzymes ACLY and ACC1 (Guo et al., 2020).

We explored the expression patterns and prognostic significance of m6A-related genes in esophageal cancer. From the TCGA database, we found that several m6A-associated genes, including YTHDF1, HNRNPC, ZC3H13, YTHDC2, and METTL14 were dysregulated in esophageal cancer compared to matched normal tissues. Our study also indicated that ALKBH5, HNRNP and WTAP correlated with the overall survival rate. Patients with high ALKBH5 expression levels had better OS, while patients with an overexpression of HNRNPC and WTAP had poor OS. HNRNPC was found to be an independent risk factor for OS through multivariate Cox regression analysis. Yan et al. (2019) demonstrated that lung cancer patients with high HNRNPC expression levels had short survival times and poor prognosis, which was consistent with our results. Huang et al. (2016a) also reported that high level of HNRNPC transcript indicated poor overall survival and progression-free survival. Therefore, the results of the expression pattern and prognostic value of the m6A-related genes indicated that HNRNPC may be the key gene in m6A modification of esophageal cancer.

Numerous studies on miRNA expression profiling have shown that the miRNA-based signature may serve as prognostic biomarkers in patients with various types of cancers (Lv et al., 2020; Tang et al., 2019). Xu et al. (2019) identified a three-miRNA signature that was significantly associated with OS and disease-specific survival (DSS) of patients with hypopharyngeal squamous cell carcinoma undergoing surgery and radiotherapy. Xie et al. (2018) established a four-miRNA signature that correlated with the survival of patients with clear cell renal cell carcinoma. Qian et al. (2019) constructed two novel miRNA-based prediction signatures of OS and recurrence-free survival (RFS), which were reliable tools to assess the prognosis of colorectal cancer patients. In addition, Xue et al. (2021) screened two independent miRNA datasets acquired from TCGA-ESCA and GSE13937 and revealed that hsa-miR-186-5p and hsa-let-7d-5p could be used as independent prognostic biomarkers for esophageal adenocarcinoma (EADC) and esophageal squamous cell carcinoma (ESCC), respectively.

In the current study, we identified 522 potential upstream miRNAs of m6A-related genes through the online tool starBase3.0. The prognostic signature was constructed from four miRNAs. The miRNA-based OS model could determine whether patients with esophageal cancer had poor- or good-OS according to their risk score. In addition, our results showed that the miRNA-based OS signature may be an independent prognostic factor of esophageal cancer. Patients in the high-risk group had a significantly poorer prognosis than those in the low-risk group.

The four prognostic miRNAs have all been previously reported to be associated with cancer. MiRNA-320c was found to inhibit tumorous behaviors of bladder cancer by targeting Cyclin-dependent kinase 6 (Wang et al., 2014). Cui et al. (2020) reported that miR-186 interacting with METTL3 contributed to the progression of hepatoblastoma by activating Wnt/β-catenin signaling pathway. Zhang et al. (2019) observed that miR-320b was downregulated in non-small cell lung cancer (NSCLC) patients and the overexpression of miR-320b inhibited cell proliferation, invasion and induced cell apoptosis in NSCLC cells. miR-320d was downregulated in the serum of hepatocellular carcinoma (HCC) patients and may be a potential non-invasive biomarker for the diagnosis and prognosis of HCC (Li et al., 2020). Each of these miRNAs appeared to have a certain degree of influence on tumor progression and the combination of these miRNAs into a signature may more effectively predict cancer outcomes. Therefore, the four-miRNA signature in our study may be used to evaluate the collective effects of these miRNAs on OS in esophageal cancer patients.

We constructed the key miRNA-m6A related gene network involving one m6A-related gene (HNRNPC) and four miRNAs (miR-186, miR-320c, miR-320d, miR-320b). Our study further validated that HNRNPC was upregulated in esophageal cancer cell lines. Functional assays showed that the knockdown of HNRNPC significantly suppressed cell proliferation, migration and invasion of KYSE30 and TE-1 cells (Fig. 6). Wu et al. (2018) suggested that the repression of HNRNPC arrested the proliferation and tumorigenesis of breast cancer cells MCF7 and T47D, which was consistent with our results. Huang et al. (2016a) demonstrated that the overexpression of HNRNPC in gastric cancer cell lines promoted chemoresistance, while its downregulation reversed chemotherapeutic resistance. Additionally, we corroborated that miR-186 directly targeted HNRNPC. Several studies have demonstrated that miR-186 acted as a tumor suppressor and was downregulated in some malignancies. He et al. (2016) reported that miR-186 suppressed the proliferation and invasion, and induced the apoptosis of esophageal squamous cell carcinoma cells. Huang et al. (2016b) showed that miR-186 could suppress cell proliferation and the migration of non–small-cell lung cancer. We demonstrated that miR-186 interacted with HNRNPC and suppressed the expression of HNRNPC. Future research should focus of exploring the mechanism of the miR-186-HNRNPC axis in esophageal cancer.

Our study had certain limitations that require clarification. Firstly, the data from our study was retrospective and should be validated in multi-center clinical trials and prospective studies. Secondly, the selected markers’ biological functions still need to be validated in future experimental studies. Despite these shortcomings, we identified a novel 4-miRNA prognostic signature and established the key miRNA-m6A related gene network in esophageal cancer for the first time. Our finding may assist in determining the prognosis and providing more accurate care for esophageal cancer patients.

## Conclusions

We successfully identified a 4-miRNA prognostic signature and established the key miRNA-m6A related gene network in patients with esophageal cancer. Future studies should focus on the molecular mechanisms of the selected markers.

## Supplemental Information

10.7717/peerj.11893/supp-1 Supplemental Information 1 Raw data.

10.7717/peerj.11893/supp-2 Supplemental Information 2 Figure6&7 raw data.

## Additional Information and Declarations

### Data Availability

The following information was supplied regarding data availability: The raw measurements are available in the Supplemental Files. The codes are available at GitHub: https://github.com/qcrcherry/m6A-miRNA.
