# Supplementary figures and images for: Network analysis of miRNA targeting m6A-related genes in patients with esophageal cancer
Source: PeerJ. 2021 Jul 29;9:e11893. doi: 10.7717/peerj.11893 (PMC8325912; doi:10.7717/peerj.11893)

|                | pvalue | Hazard ratio       |
|----------------|--------|--------------------|
| hsa-mir-186    | 0.018  | 1.872(1.115–3.145) |
| hsa-mir-495    | 0.213  | 0.847(0.653–1.100) |
| hsa-mir-320a   | 0.683  | 0.891(0.512–1.551) |
| hsa-mir-320b-1 | 0.606  | 1.141(0.691–1.884) |
| hsa-mir-320c-1 | 0.008  | 0.451(0.251–0.809) |
| hsa-mir-548o   | 0.609  | 1.232(0.554–2.735) |
| hsa-mir-320d-1 | 0.081  | 1.942(0.922–4.090) |

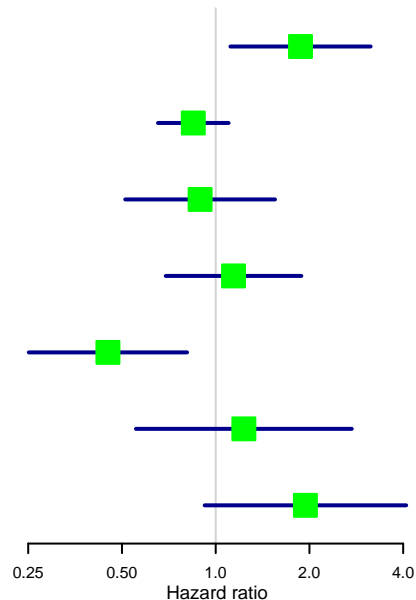

Supplement: Supplemental Information 1 [file peerj-09-11893-s001.zip › Supplemental Materials/10.Cox analysis of miRNA/multivariate cox analysis-forest.pdf]

|                | pvalue | Hazard ratio       |
|----------------|--------|--------------------|
| hsa-mir-186    | 0.003  | 1.836(1.223–2.758) |
| hsa-mir-495    | 0.751  | 0.963(0.760–1.218) |
| hsa-mir-320a   | 0.351  | 1.179(0.834–1.667) |
| hsa-mir-320b-1 | 0.394  | 1.141(0.843–1.545) |
| hsa-mir-320c-1 | 0.053  | 0.620(0.382–1.007) |
| hsa-mir-548o   | 0.596  | 1.217(0.589–2.514) |
| hsa-mir-320d-1 | 0.182  | 1.521(0.822–2.816) |

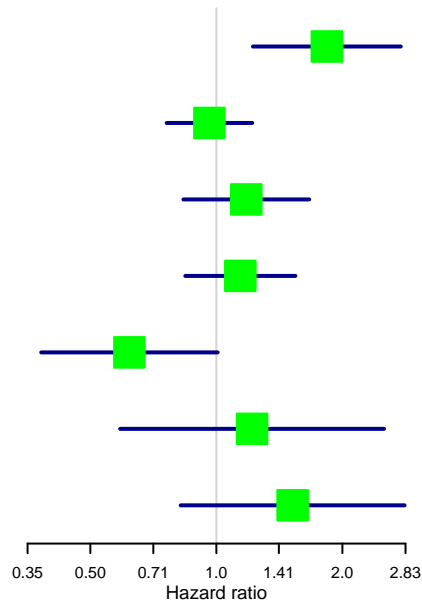

Supplement: Supplemental Information 1 [file peerj-09-11893-s001.zip › Supplemental Materials/10.Cox analysis of miRNA/univariate cox analysis-forest.pdf]

Partial Likelihood Deviance

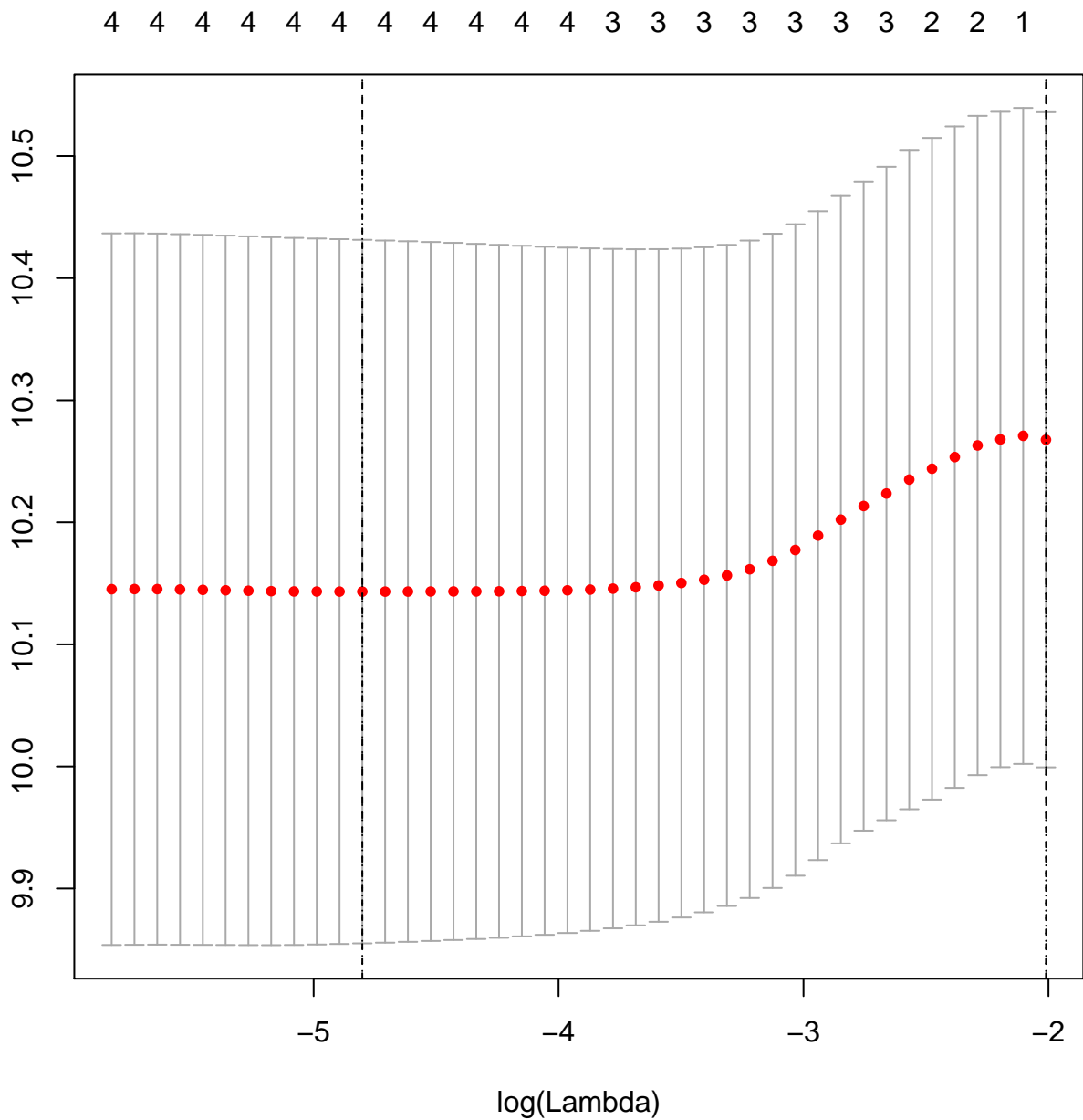

Supplement: Supplemental Information 1 [file peerj-09-11893-s001.zip › Supplemental Materials/11.lasso regression/cvfit.pdf]

Coefficients

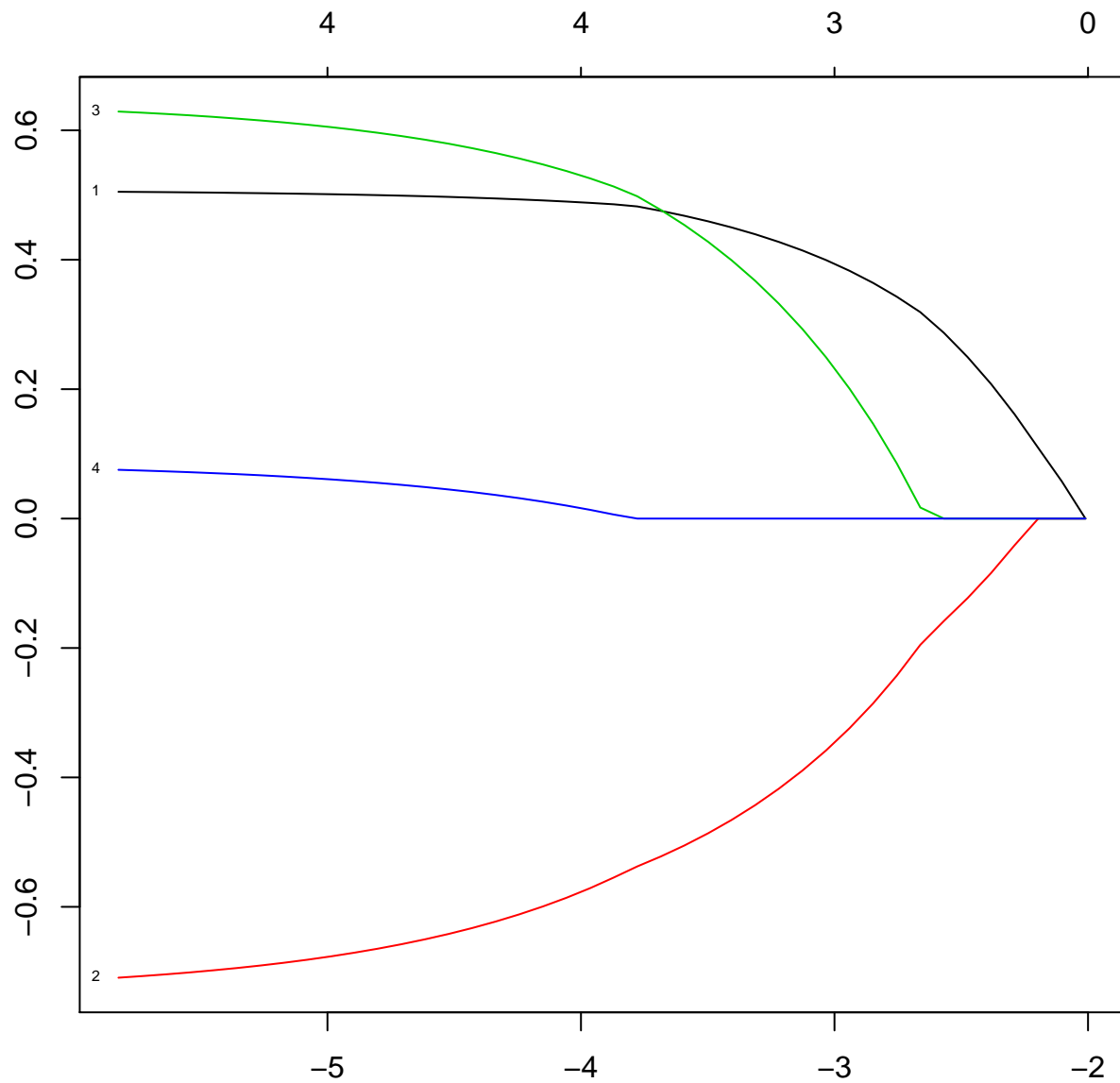

Log Lambda

Supplement: Supplemental Information 1 [file peerj-09-11893-s001.zip › Supplemental Materials/11.lasso regression/lambda.pdf]

**Survival curve ( $p=4.516e-04$ )**

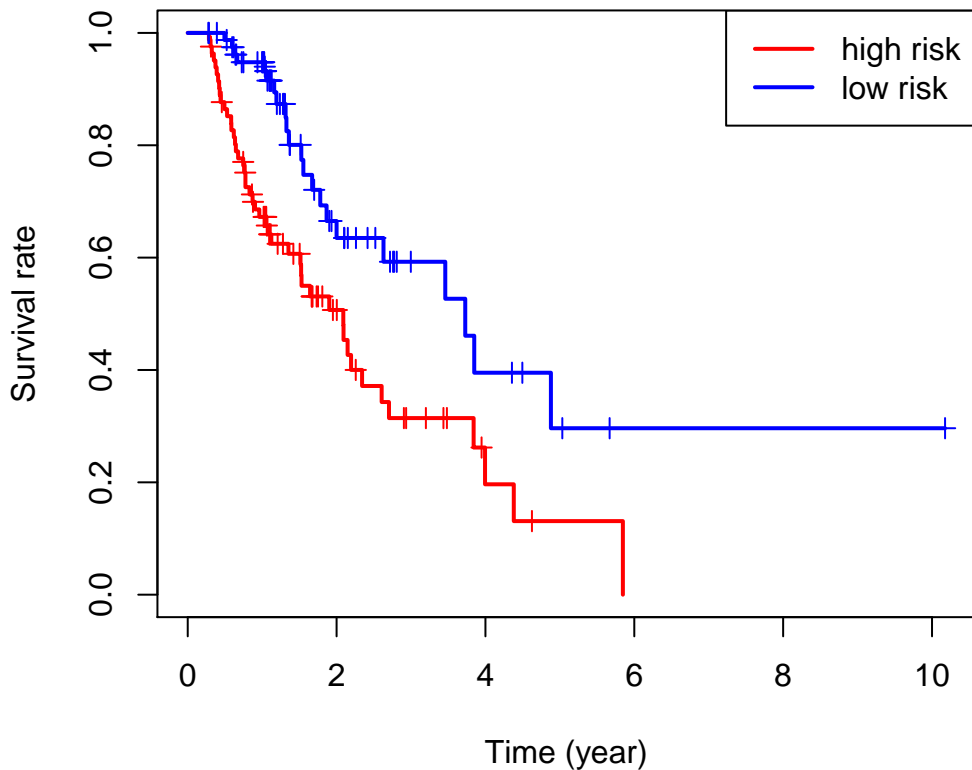

Supplement: Supplemental Information 1 [file peerj-09-11893-s001.zip › Supplemental Materials/12.Kaplan¿CMeier OS curve for patients assigned to high-risk and low-risk groups/survival.pdf]

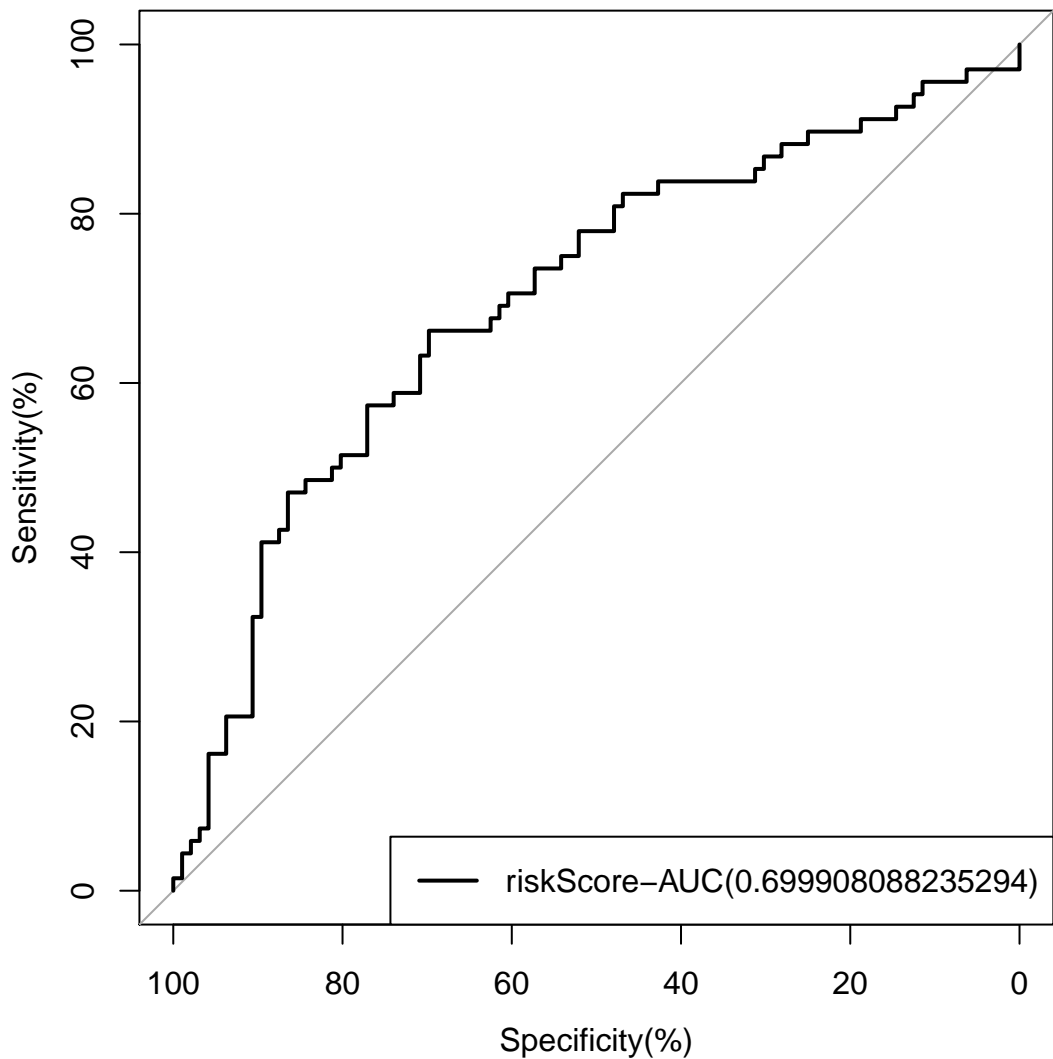

Supplement: Supplemental Information 1 [file peerj-09-11893-s001.zip › Supplemental Materials/13.ROC curve of the risk signature/roc.pdf]

|           | pvalue | Hazard ratio       |
|-----------|--------|--------------------|
| age       | 0.311  | 0.987(0.962–1.012) |
| gender    | 0.383  | 1.901(0.449–8.050) |
| grade     | 0.983  | 1.006(0.603–1.679) |
| stage     | 0.048  | 1.516(1.003–2.292) |
| T         | 0.392  | 1.180(0.808–1.723) |
| M         | 0.209  | 1.844(0.710–4.784) |
| N         | 0.004  | 1.873(1.227–2.858) |
| riskScore | 0.043  | 1.013(1.000–1.026) |

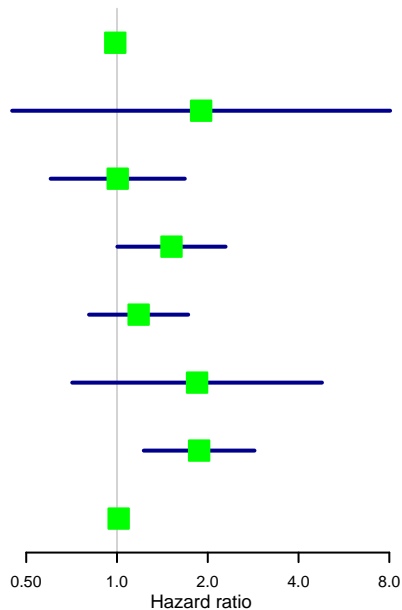

Supplement: Supplemental Information 1 [file peerj-09-11893-s001.zip › Supplemental Materials/15.Univariate Cox regression analyse of the association between clinicopathological factors (including the risk score) and overall survival of esophageal cancer patients/forest.pdf]

|           | pvalue | Hazard ratio        |
|-----------|--------|---------------------|
| age       | 0.487  | 0.989(0.958–1.021)  |
| gender    | 0.160  | 3.110(0.640–15.117) |
| grade     | 0.087  | 0.532(0.258–1.096)  |
| stage     | 0.757  | 0.837(0.271–2.580)  |
| T         | 0.453  | 1.288(0.665–2.497)  |
| M         | 0.757  | 1.322(0.225–7.761)  |
| N         | 0.025  | 1.986(1.092–3.611)  |
| riskScore | 0.011  | 1.023(1.005–1.041)  |

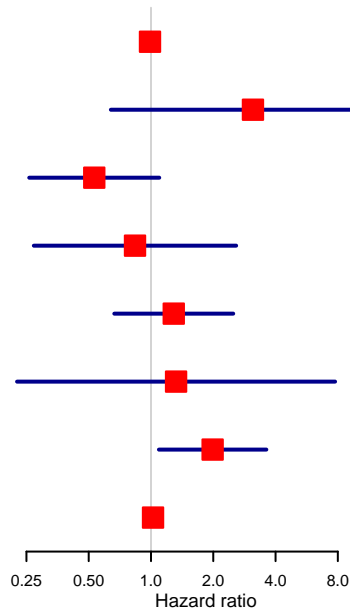

Supplement: Supplemental Information 1 [file peerj-09-11893-s001.zip › Supplemental Materials/16.Multivariate Cox regression analyse of the association between clinicopathological factors (including the risk score) and overall survival of esophageal cancer patients/forest.pdf]

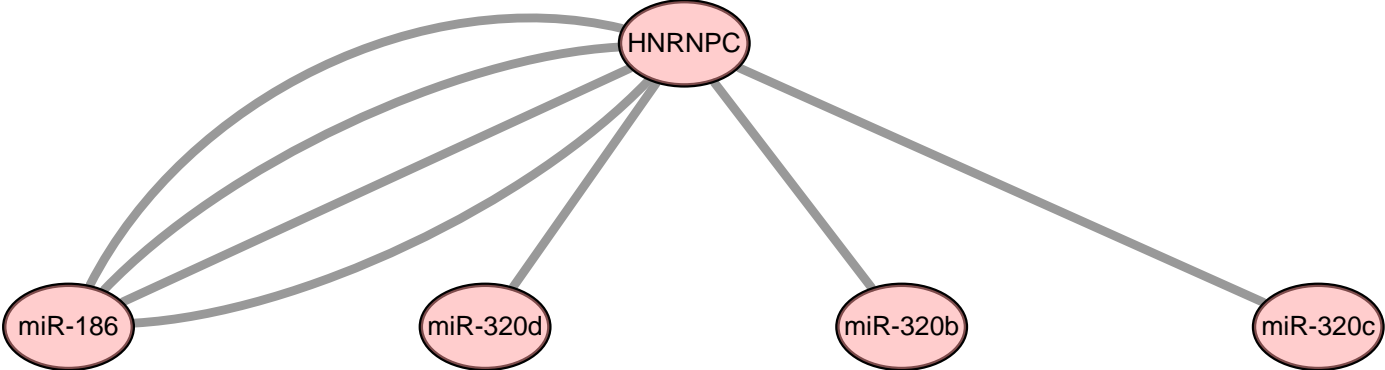

Supplement: Supplemental Information 1 [file peerj-09-11893-s001.zip › Supplemental Materials/17.The key miRNA-m6A related gene network/The key miRNA-m6A related gene network.pdf]

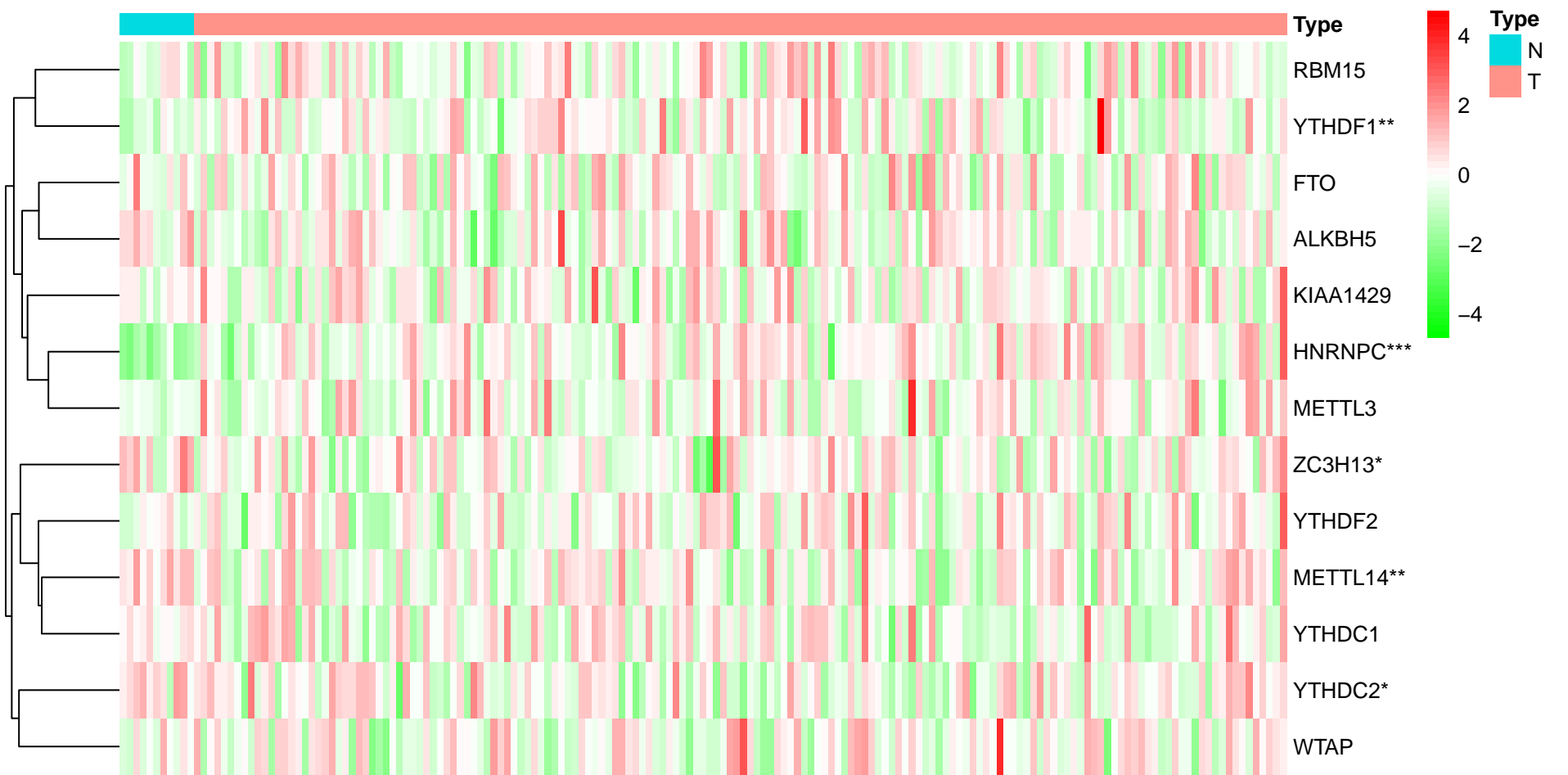

Supplement: Supplemental Information 1 [file peerj-09-11893-s001.zip › Supplemental Materials/2.Heat map of M6A gene expression/heatmap-TCGA.pdf]

Gene expression

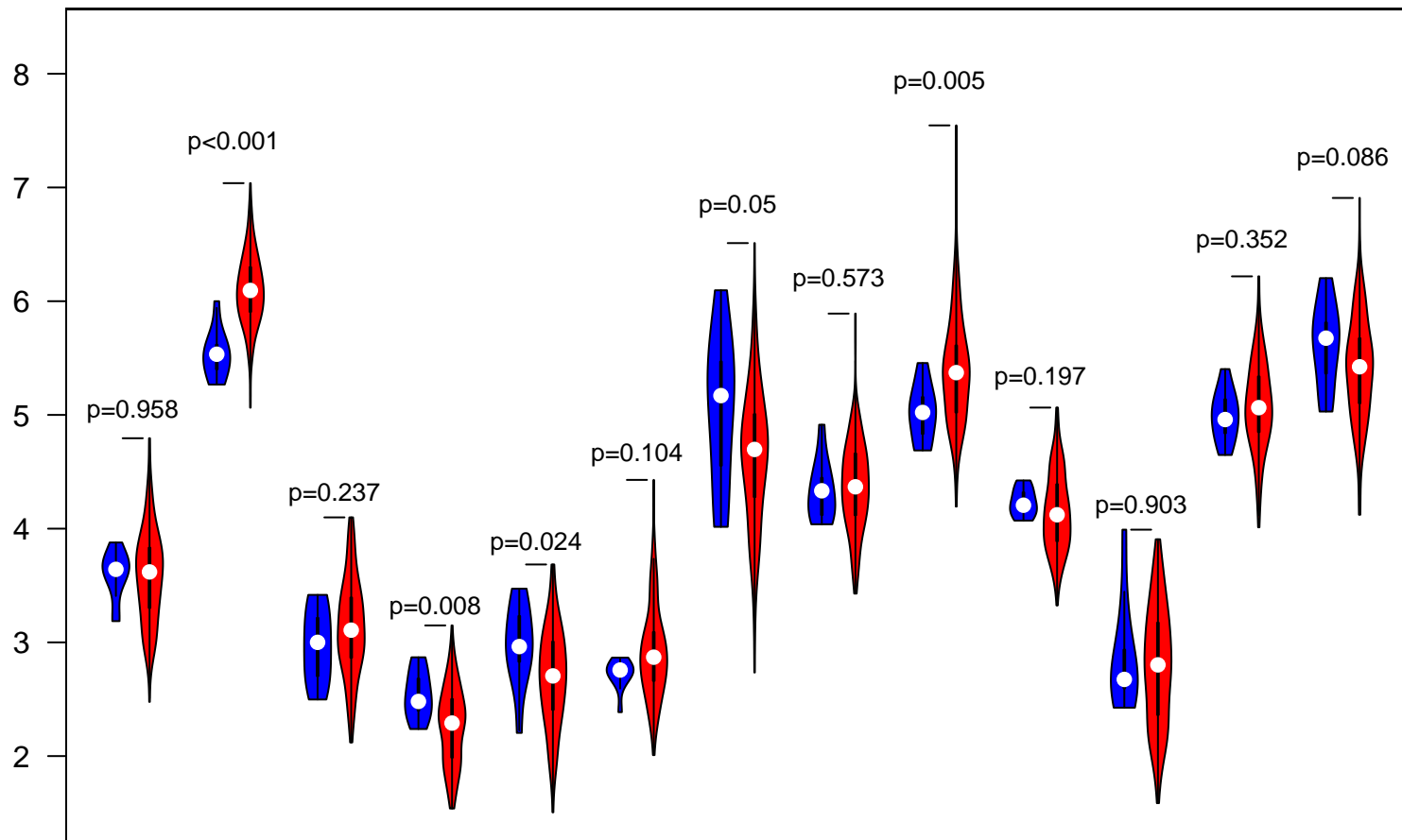

Supplement: Supplemental Information 1 [file peerj-09-11893-s001.zip › Supplemental Materials/3.vioplot of M6A gene expression/vioplot-tcga.pdf]

**Survival curve (p=0.007)**

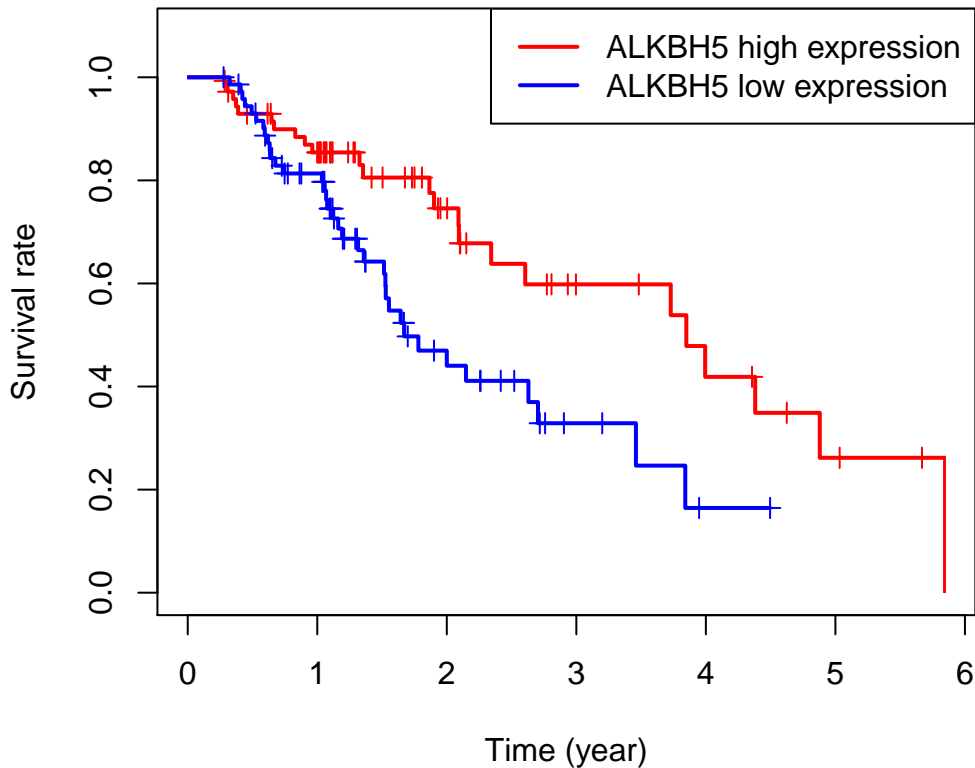

Supplement: Supplemental Information 1 [file peerj-09-11893-s001.zip › Supplemental Materials/4.survival analysis of m6A gene/ALKBH5.survival.pdf]

**Survival curve (p=0.034)**

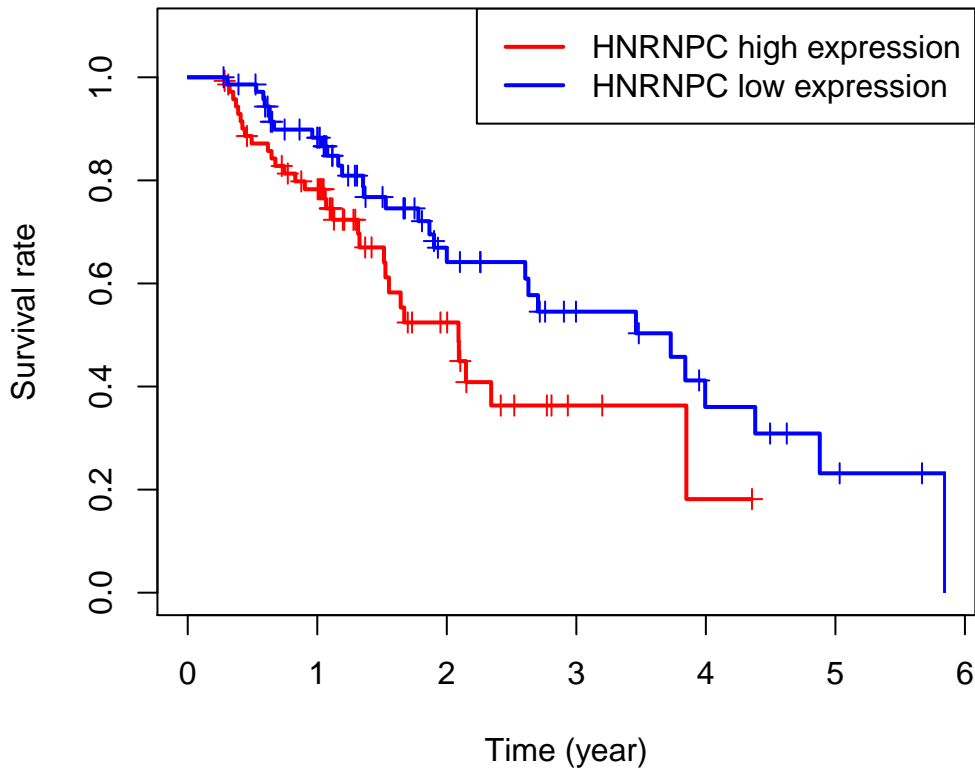

Supplement: Supplemental Information 1 [file peerj-09-11893-s001.zip › Supplemental Materials/4.survival analysis of m6A gene/HNRNPC.survival.pdf]

**Survival curve (p=0.047)**

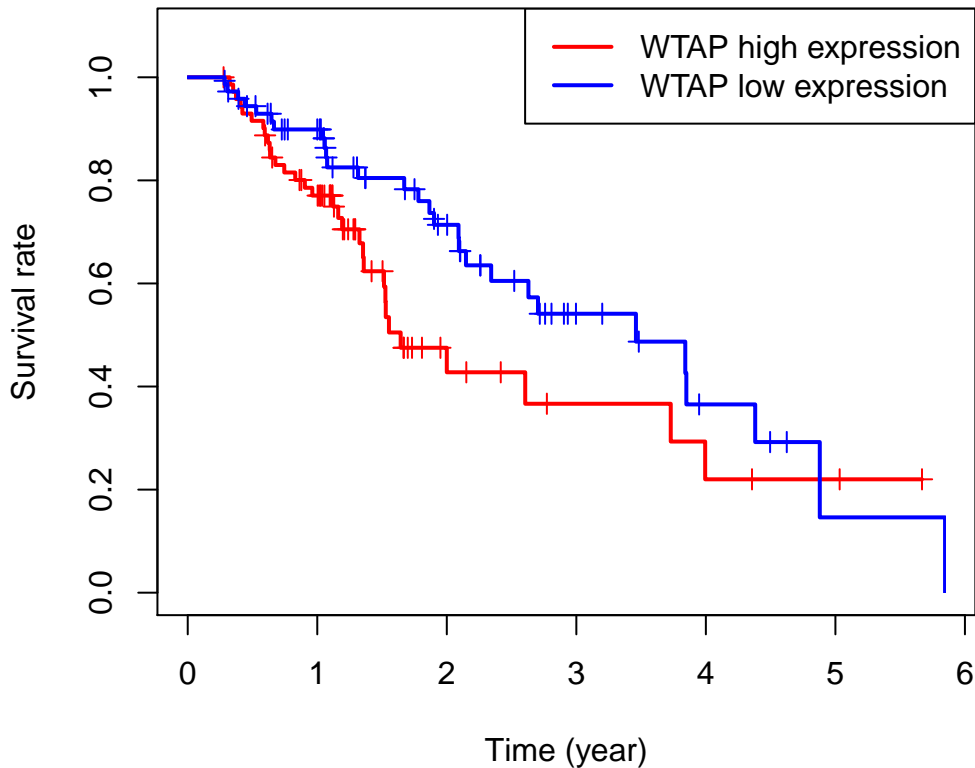

Supplement: Supplemental Information 1 [file peerj-09-11893-s001.zip › Supplemental Materials/4.survival analysis of m6A gene/WTAP.survival.pdf]

|          | pvalue | Hazard ratio        |
|----------|--------|---------------------|
| KIAA1429 | 0.076  | 1.916(0.934–3.931)  |
| HNRNPC   | 0.021  | 4.171(1.244–13.986) |
| RBM15    | 0.934  | 1.033(0.474–2.254)  |
| METTTL14 | 0.015  | 0.279(0.100–0.778)  |
| YTHDC2   | 0.444  | 1.374(0.609–3.099)  |
| METTTL3  | 0.468  | 0.723(0.301–1.736)  |
| ZC3H13   | 0.390  | 1.262(0.742–2.147)  |
| WTAP     | 0.592  | 1.253(0.549–2.864)  |
| YTHDF1   | 0.956  | 1.019(0.525–1.979)  |
| YTHDC1   | 0.197  | 1.976(0.703–5.553)  |
| FTO      | 0.551  | 0.829(0.448–1.535)  |
| YTHDF2   | 0.437  | 0.673(0.248–1.829)  |
| ALKBH5   | 0.007  | 0.371(0.181–0.760)  |

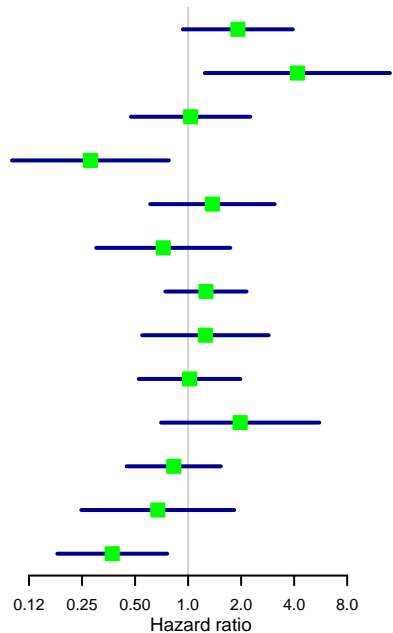

Supplement: Supplemental Information 1 [file peerj-09-11893-s001.zip › Supplemental Materials/5.Cox analysis of m6A gene/multivariate analysis-forest.pdf]

|          | pvalue | Hazard ratio       |
|----------|--------|--------------------|
| KIAA1429 | 0.449  | 1.275(0.680–2.391) |
| HNRNPC   | 0.103  | 2.059(0.864–4.911) |
| RBM15    | 0.867  | 0.949(0.513–1.755) |
| METTTL14 | 0.063  | 0.473(0.214–1.043) |
| YTHDC2   | 0.742  | 0.900(0.479–1.689) |
| METTTL3  | 0.825  | 1.079(0.548–2.124) |
| ZC3H13   | 0.374  | 1.198(0.804–1.786) |
| WTAP     | 0.353  | 1.400(0.689–2.848) |
| YTHDF1   | 0.670  | 0.877(0.480–1.602) |
| YTHDC1   | 0.515  | 1.301(0.589–2.876) |
| FTO      | 0.369  | 0.780(0.454–1.341) |
| YTHDF2   | 0.267  | 0.673(0.334–1.355) |
| ALKBH5   | 0.001  | 0.361(0.195–0.668) |

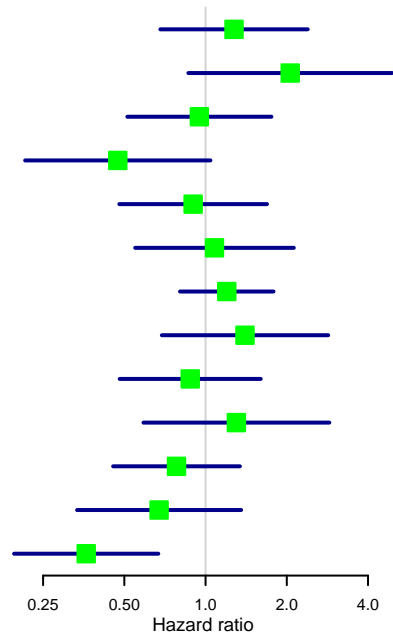

Supplement: Supplemental Information 1 [file peerj-09-11893-s001.zip › Supplemental Materials/5.Cox analysis of m6A gene/univariate analysis-forest.pdf]

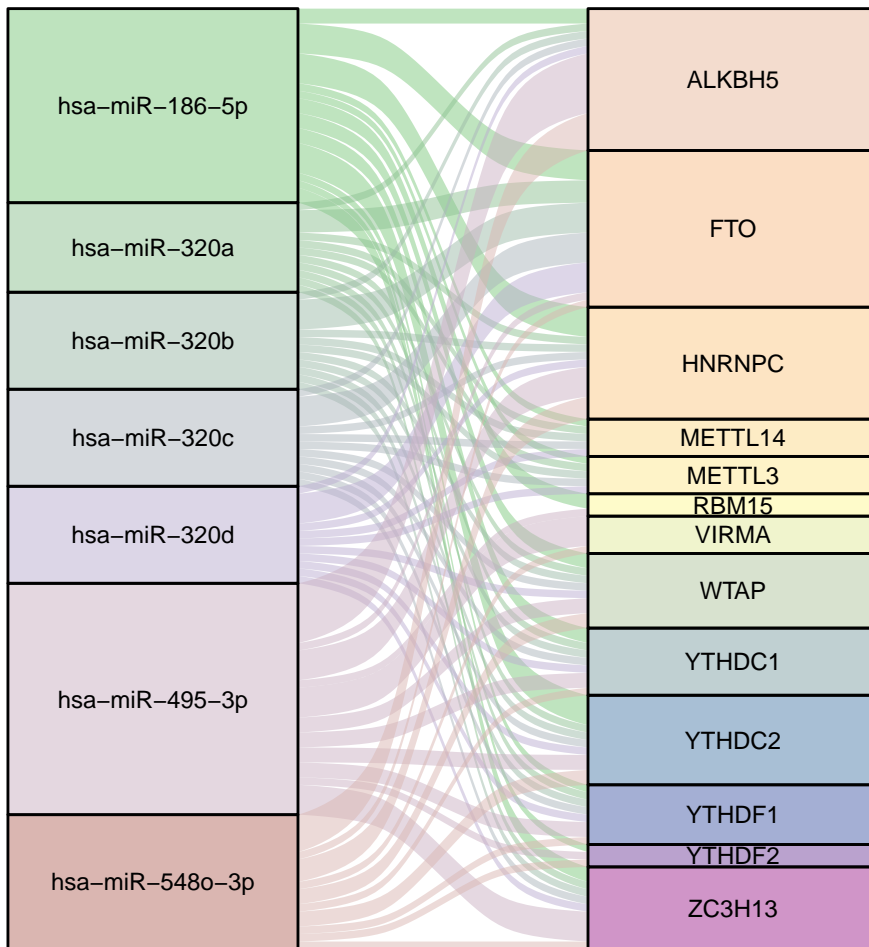

miRNA

m6A Gene

Supplement: Supplemental Information 1 [file peerj-09-11893-s001.zip › Supplemental Materials/6.Starbase predicts upstream miRNAs/The relationship between the 13 M6A genes and their corresponding miRNAs.pdf]

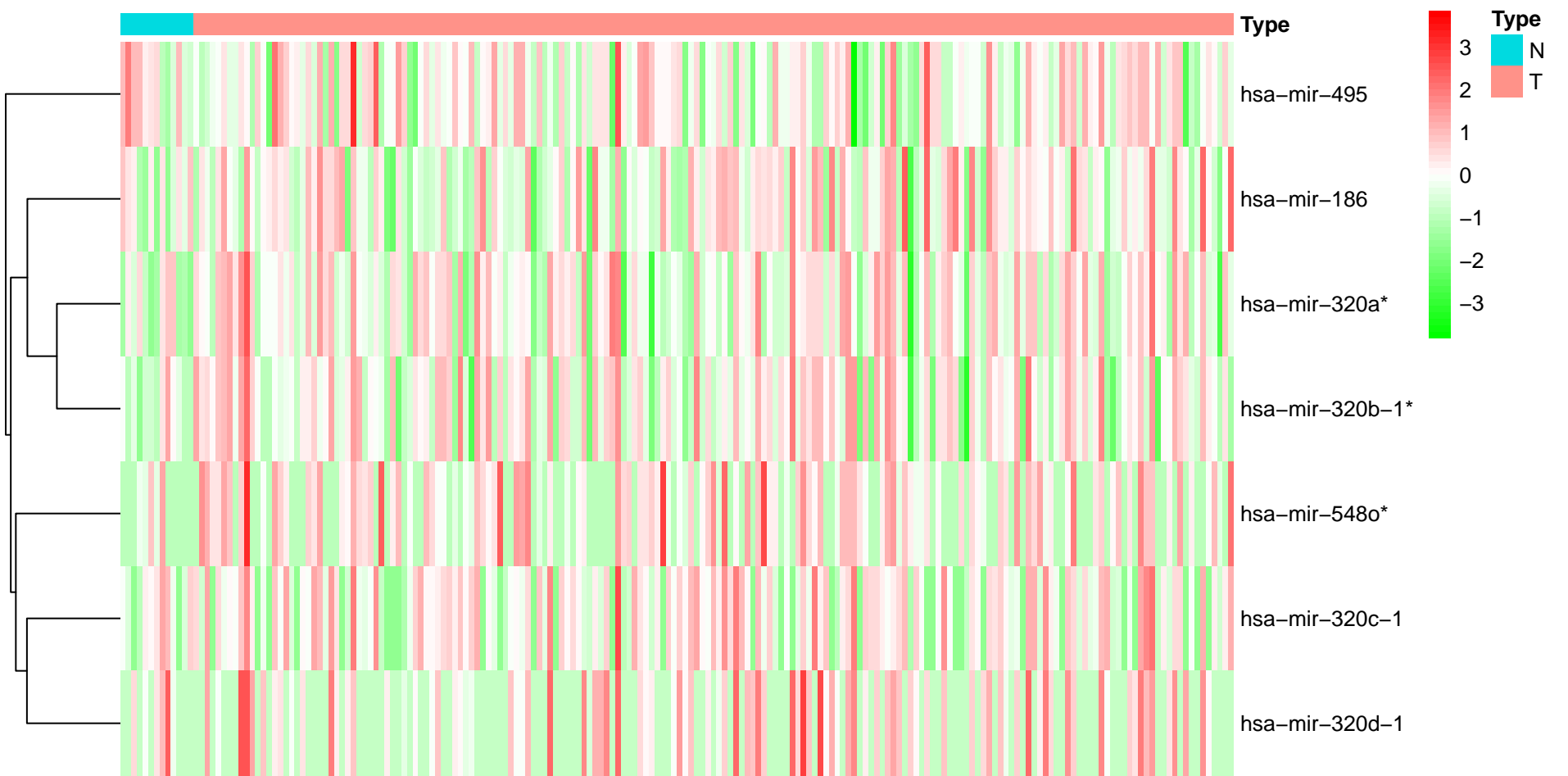

Supplement: Supplemental Information 1 [file peerj-09-11893-s001.zip › Supplemental Materials/8.Heat map of miRNA expression/heatmap.pdf]

miRNA expression

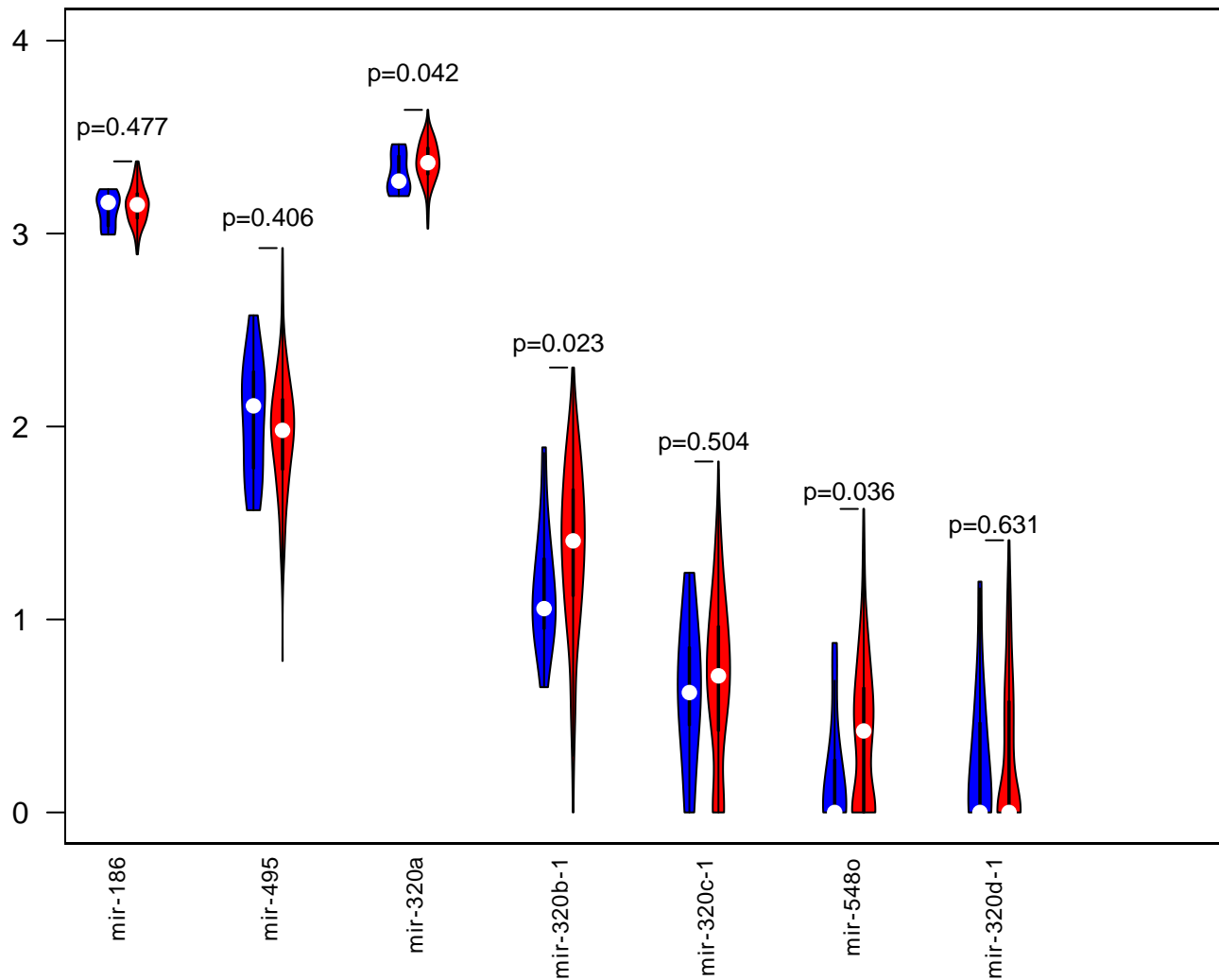

Supplement: Supplemental Information 1 [file peerj-09-11893-s001.zip › Supplemental Materials/9.vioplot of miRNA expression/vioplot.pdf]
